# Are Physicians in Saudi Arabia Ready for Patients with an Insulin Pump? An Examination of Physician Knowledge and Attitude

**DOI:** 10.3390/ijerph17249394

**Published:** 2020-12-15

**Authors:** Aqeel Alaqeel, Abdulaziz Almushaigeh, Muna Almijmaj, Raghad Almesned, Mohammed Alsuhaibani

**Affiliations:** 1 Department of Pediatrics, College of Medicine, Qassim University, Qassim 51452, Saudi Arabia; msuhaibani@qumed.edu.sa; 2 Medical Intern, College of Medicine, Qassim University, Qassim 51452, Saudi Arabia; Abdulazizmoshaigeh@gmail.com (A.A.); m.ab.almijmaj@gmail.com (M.A.); Raghadalmisned@gmail.com (R.A.)

**Keywords:** insulin pump therapy, diabetes, knowledge, attitude, physicians

## Abstract

Aims: The use of insulin pump therapy in patients with diabetes continues to expand worldwide. Although insulin pumps have been demonstrated to be successful and safe, physicians’ insufficient knowledge may carry a risk to the patients using insulin pumps. This study aimed to assess the attitude and knowledge among physicians in Saudi Arabia regarding insulin pump therapy. Methods: Three hundred and seven physicians, including 82 family physicians, 76 pediatricians, 48 internists, 27 pediatric endocrinologists, 17 adult endocrinologists, and 57 physicians from other specialties, completed a questionnaire that evaluated their knowledge and attitude toward insulin pump therapy. Results: Among the evaluated physicians, 56.7% had poor knowledge level, while 53.4% had positive attitude. Statistical tests revealed that older age, years of practice, consultancy, and endocrinology specialty were the influential factors of knowledge (*p* < 0.001). Non-endocrinologists demonstrated poor knowledge despite seeing patients with insulin pumps; however, those who had previously cared for such patients scored significantly higher knowledge scores. Conclusions: There was a significant lack of knowledge among physicians regarding insulin pump therapy; however, the perceived attitude of physicians toward this therapy was deemed positive. These findings support the implementation of insulin pump education programs.

## 1. Introduction

Diabetes management has advanced recently in different aspects, particularly for patients who require insulin therapy. The main advances include new insulin analogs, technology for monitoring glucose readings, and insulin delivery. Continuous subcutaneous insulin infusion (insulin pump) is considered one of the preferred insulin delivery modalities that started in the late 1970s and continued to develop until it reached a technology level where it mimics normal insulin secretion [1,2,3]. The insulin pump is a small dynamic device that can deliver rapid-acting insulin throughout the day to manage glycemic levels [1]. Furthermore, various studies have proven the superiority of insulin pump therapy across all age groups of patients with diabetes regarding glycemic control, duration of normoglycemia, incidence of severe hypoglycemia, quality of life, and lower rate of long-term complications [3,4,5]. On the other hand, patients on insulin pump therapy do not receive long-acting insulin. Therefore, once insulin delivery is interrupted for several hours due to pump malfunction, infusion set problems, or manual insulin suspension without giving alternative insulin through injection, acute hyperglycemia, and diabetic ketoacidosis may occur [3].

In the United States, approximately 400,000 patients with diabetes use insulin pumps. In Saudi Arabia, it is not known how many patients there are on insulin pumps; however, a recent cross-sectional study shows that 9% of children with type 1 diabetes use insulin pump therapy [6,7,8]. With the increasing prevalence of diabetes, both worldwide and nationally, and the continuous technology advancement of the insulin pump that improves glycemic control, we believe that it will gain more popularity in the coming years [1,9].

The potential challenges in regard to insulin pump therapy in Saudi Arabia include the following. First, insulin pumps are prescribed in tertiary centers by qualified diabetes teams. However, patients from outside the city may visit hospitals in their living area, and physicians are obligated to provide adequate care and deal with the insulin pump. Second, based on our practice, patients with diabetes can get insulin pumps without a prescription from online markets outside the country. Third, in addition to endocrinologists, physicians of different specialties, such as family physicians, internists, and pediatricians, are included in the management of patients with diabetes [10].

With the increasing use of insulin pump therapy, physicians will need to deal with patients’ insulin pumps at inpatient, outpatient, and emergency visits, and may find themselves facing difficulties related to their lack of knowledge regarding the management of insulin pumps, which may negatively affect the patients’ outcomes. To date, to the best of our knowledge, there has been no study on a national or international level to evaluate the knowledge and attitude regarding insulin pumps in physicians of different specialties who may provide care to adults and children with diabetes. In the present study, we assessed the knowledge and attitude of physicians in Saudi Arabia regarding insulin pump therapy.

## 2. Materials and Methods

### 2.1. Ethical Considerations

Approval for this study was obtained from the Committee of Bioethics of the Qassim Health Affairs Directorate and the Ministry of Health (H-04-Q-001). All participants provided written informed consent before filling the survey.

### 2.2. Study Design and Participants

This was a cross-sectional study conducted between 1 June 2020 and 30 August 2020 in Saudi Arabia. Participants included Saudi and non-Saudi physicians who worked in hospitals in different Saudi regions. Physician specialties targeted by this study included pediatrics, internal medicine, family medicine, and endocrinology. Physicians with different positions were included, such as residents, specialists/registrars, and consultants. The survey was distributed to 380 physicians in scientific social media groups in the country. The groups contain all specialties, including diabetologists. The invitation was posted to fill the survey if the participants are taking care of patients with diabetes. The authors explain the survey’s purpose privately and invited them to fill it. The authors sent two reminders within one month for those who agreed to participate. Out of 380 physicians, 307 participated, with a response rate of 80%.

### 2.3. Survey Design and Distribution

For the survey in this study, we used a questionnaire that was created by a similar study based on the American Diabetes Association and National Institute for Health and Care Excellence, they have validated the questionnaire by 7 experts [11]. In addition, we performed minor modifications to target physicians and diabetologists who may frequently deal with the insulin pump. The modified survey and grading of knowledge and attitude were revised by two independent endocrinologists who are expert in insulin pump therapy and research. The survey was pretested before it was distributed to the participants. We created a Google form containing the survey and distributed it to the participants through social media applications.

The questionnaire contained 28 questions organized in three sections. The first section included eight questions on demographic data, including age, gender, nationality, residency, years of experience in the medical filled, specialty, current position, and availability of endocrinologists in the workplace. The second section included six questions related to the attitude of physicians toward insulin pump therapy, such as frequency of dealing with insulin pumps, their opinion about the importance of gaining necessary information, educational program to ensure the safety of dealing with insulin pumps, the impact of insulin pump therapy on patients, criteria for patient selection, and barriers to use insulin pump therapy. The third section comprised 14 questions related to the physicians’ knowledge on insulin pump therapy. The questions covered the basic information, including pump function, technique, type of insulin commonly used, therapy regimen, patients’ eligibility, change of the infusion set, as well as risks in case of insulin pump interruption.

### 2.4. Scoring

The physicians’ knowledge regarding insulin pump therapy was evaluated as follows. For each of the 14 questions, a score of 1 was assigned for correct answers and a score of 0 for incorrect answers. The total knowledge score, calculated as the sum of all individual scores, ranged from 0 to 14 points, with higher scores indicating greater knowledge regarding insulin pump therapy. Using cutoff points at 50% and 75% of the total score, participants were classified as having poor (0–6), average (7–10), or good knowledge (11–14).

For evaluation of the attitude regarding insulin pump therapy, scores of 1 to 5 were assigned for the answers to the six questions based on a five-point Likert scale ranging from “strongly disagree” (score 1) to “strongly agree” (score 5). The total attitude score, calculated as the sum of all individual scores, ranged from 5 to 30 points, with higher scores indicating a more positive attitude regarding insulin pump therapy. Using cutoff points at 50% and 75% of the total score, participants were classified as having a negative (5–15), neutral (16–22), or positive attitude (23–30).

### 2.5. Statistical Analysis

Descriptive statistics were demonstrated using numbers, percentages, and mean ± standard deviation, whenever appropriate. Between-group comparisons were performed using the Mann–Whitney U test and the Kruskal–Wallis test. Between significant results, post hoc analyses were performed using Bonferroni test.

Normality of data distribution was assessed using Shapiro–Wilk’s test. Pearson’s correlation test was used to determine the linear relationship between the knowledge and attitude scores. All statistical analyses were performed using IBM SPSS Statistics V21.0 (IBM Corp., Armonk, NY, USA). A *p*-value of < 0.05 was considered as statistically significant.

## 3. Results

### 3.1. Participants’ Characteristics

A total of 307 physicians participated in the survey. As shown in Table 1, the most common age group was 21–30 years (51.1%). The most frequent specialties were family medicine (26.7%) and pediatrics (24.8%). With respect to their current position, nearly half of all physicians (47.6%) were residents, followed by consultants (33.6%) and specialists/registrars (22.1%).

### 3.2. Knowledge Regarding Insulin Pump Therapy

The results of the assessment of the physicians’ knowledge regarding insulin pump therapy are presented in Table 2.

### 3.3. Attitude toward Insulin Pump Therapy

In the assessment of the physicians’ attitude toward insulin pump therapy (Table 3), nearly half (48.5%) strongly agreed that physicians should know the basic information and understand the primary principles of insulin pump therapy. Furthermore, 66.8% of physicians strongly agreed that each hospital institution should have a structured diabetes program for patients who are using insulin pumps.

### 3.4. Comparison of the Knowledge and Attitude Scores Based on the Participants’ Characteristics

When comparing the knowledge and attitude scores based on the sociodemographic characteristics of the participating physicians (Table 4), we observed that older age was associated with greater knowledge (*p* < 0.001) and attitude (*p* = 0.049) scores. Furthermore, the knowledge of pediatric endocrinology consultants was statistically significantly higher than that to other consultants (F = 12.881; *p* < 0.001), respectively. In addition, we found that a longer working experience was significantly associated with a higher knowledge score (*p* < 0.001). Moreover, non-endocrinologists who had seen patients with insulin pumps 2–3 times per month had significantly greater knowledge than those who had not provided care to such patients (Supplementary Materials Tables S1–S3). There were no significant differences in the knowledge and attitude scores according to other variables, such as gender, nationality, residence region (Supplementary Materials Table S4).

**Figure 1:**
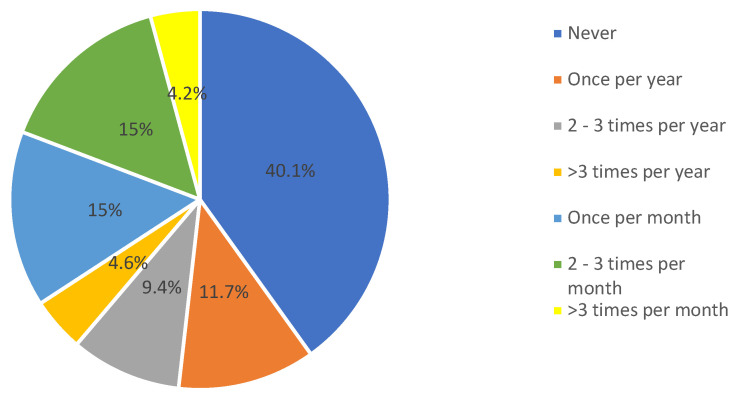
How often do you see a patient on insulin pump?

Figure 1 depicts the proportion of physicians who had seen a patient with insulin pump. It was revealed that 30% of them had seen a patient with insulin pump in 2–3 times per month or once per month, while 40.1% reported that they have not seen any patients using insulin pump.

**Figure 2:**
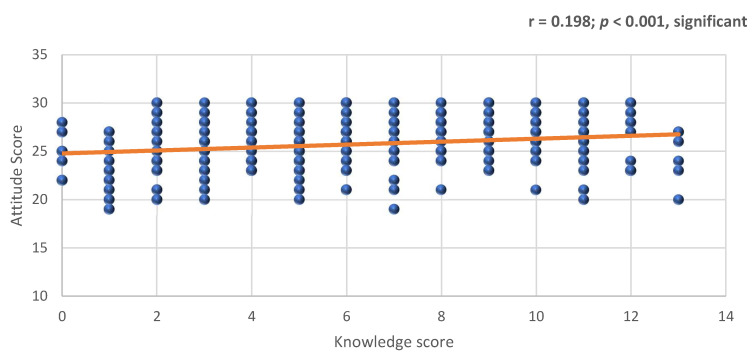
Correlation (Pearson-R) between the mean score of knowledge and attitude.

In Figure 2, we found that the correlation between knowledge and attitude was positively statistically significant (r = 0.198; *p* < 0.001).

## 4. Discussion

In the present study, we assessed the knowledge and attitude of physicians in Saudi Arabia regarding insulin pump therapy. Our results indicate a significant lack of knowledge among physicians regarding insulin pump therapy; however, the perceived attitude of physicians toward this therapy was deemed positive. Older age, years of practice, consultancy, and endocrinology specialty were found to be the influential factors of knowledge.

The deficiency in basic knowledge on insulin pumps among healthcare providers could lead to potential mishandling of insulin pumps among patients with diabetes. Only one study conducted in Riyadh, Saudi Arabia, discussed the level of knowledge and the attitude among healthcare providers in Saudi Arabia regarding insulin pump therapy [11]. The present study included participants from different specialties, which is important because the management of patients with diabetes in the country is usually done under different specialties [10]. Moreover, this study provides important information from most regions in Saudi Arabia regarding the assessment of knowledge and attitude toward insulin pump therapy. We compared the results of our study to the results of the original study “The attitude and basic knowledge of insulin pumps therapy among health care providers of King Saud University Medical City”, in which they compared the knowledge and attitude toward insulin pump among healthcare providers, including: physicians, pharmacist and nurses. They found that most of their participants have poor knowledge in which they lack a basic information regarding the insulin pump. Similarly, our study showed that most of the participants (56.7%) exhibited poor knowledge. However, this study confirms that Endocrinologists score higher knowledge. Both the current study and the original study demonstrate an acceptable level of attitude toward the insulin pump.

Our study showed that most of the participants (56.7%) exhibited poor knowledge. Such a low level of knowledge could be explained by less training and exposure to insulin pumps. On the other hand, endocrinologists displayed the highest level of knowledge, likely because they are most updated regarding such devices by attending conferences and are the target population of insulin pump manufacturers’ advertising and sales campaigns. Additionally, endocrinologists have a higher level of education regarding diabetes.

It has been reported that hospitalized patients using an insulin pump prefer to continue wearing their pump during the hospitalization period [12]. Thus, it is a prerequisite that physicians responsible for these patients’ care be experienced in insulin pumps in order to guarantee the patients’ safety and well-being, since managing an insulin pump by a physician with poor knowledge can lead to serious complications, such as diabetic ketoacidosis or severe hypoglycemia [13]. Moreover, unqualified physicians may refuse to prescribe an insulin pump to patients who may benefit from it.

Additionally, our findings showed that a longer experience in the medical field and being a consultant or specialist were associated with a higher level of knowledge regarding dealing with patients on insulin pump therapy. This finding may simply reflect the fact that these physicians have more experience, adequate exposure to more patients, and thus, more opportunities to understand insulin pumps and their effect on patients. Furthermore, they tend to be more well-informed on updated research and medical literature related to diabetes treatment.

Overall, most of our participants from different specialties showed a positive attitude toward insulin pumps. Due to the scarcity of studies on this topic, we speculated that this finding is also important as it showed the agreement between knowledge and attitude, which could be the basis for future investigations.

According to our results, endocrinologists are the most qualified physicians to deal with patients using insulin pumps, as they showed higher scores in all the different aspects of our survey. That said, it should be noted that internists, pediatricians, and family medicine physicians in Saudi Arabia are still not ready to handle patients with diabetes using insulin pumps, as most of them demonstrated an insufficient level of knowledge, which can greatly affect the management of these patients. This particular situation is made even more complicated with the apparent shortage of specialized endocrinologists throughout Saudi Arabia, as approximately 15% of the participating physicians reported a lack of an endocrinologist at their current workplace.

Therefore, we recommend that the Ministry of Health of Saudi Arabia takes appropriate steps to increase the number of qualified physicians to deal with patients on insulin pump therapy, particularly considering the constantly increasing number of diabetes cases in Saudi Arabia every year. In order to improve the level of knowledge, we suggest establishing training programs focusing on dealing with patients using insulin pumps. Additionally, due to a lack of studies reviewing the management of these patients in Saudi Arabia, we recommend future studies focusing on the difficulties that healthcare providers face with handling patients using insulin pumps.

Finally, the management of patients with diabetes in the holy month of Ramadan is challenging for patients, families and healthcare providers. This challenge is remarkable for patients on insulin therapy, including insulin pump. There are several reports from Muslim countries addressing the use of insulin pump therapy during fasting in the month of Ramadan with mixed results. However, the patients may have a higher risk of developing hypoglycemia and ketosis. Therefore, it is recommended to perform pre-Ramadan counseling for the patients and adjusting the insulin pump settings. Measuring physicians’ knowledge and attitude in this subject was not tested in the study but can be addressed in future research [14,15].

There are several limitations in our study. It was a cross-sectional study and the survey was conducted online due to the COVID-19 circumstances. There is also the possibility of recall bias as our questionnaire contained some memory-based questions.

## 5. Conclusions

Our results indicate a significant lack of knowledge among physicians regarding insulin pump therapy; however, the perceived attitude of physicians toward this therapy was deemed positive. Therefore, we recommend that the Ministry of Health of Saudi Arabia takes appropriate steps to increase the number of qualified physicians to deal with patients on insulin pump therapy, particularly considering the constantly growing prevalence of diabetes cases in Saudi Arabia every year. In order to improve the level of knowledge, we suggest establishing insulin pump training programs at the level of Saudi Ministry of Health and/or Saudi Endocrine Society. Furthermore, we recommend the physicians to gain knowledge in regard to insulin pump therapy through self-study, reading updated researches, courses and online videos.

## Figures and Tables

**Table 1:** Sociodemographic characteristics of physicians (n = 307).

Study Variables	N (%)
Age group	
21–30 years	157 (51.1%)
31–40 years	107 (34.9%)
41–50 years	28 (9.1%)
>50 years	15 (4.9%)
Gender	
Male	229 (74.6%)
Female	78 (25.4%)
Nationality	
Saudi	287 (93.5%)
Non-Saudi	20 (6.5%)
Region of residence	
Central region	170 (55.4%)
Northern region	20 (6.5%)
Western region	72 (23.5%)
Southern region	18 (5.9%)
Eastern region	27 (8.8%)
Specialty	
Pediatrician	76 (24.8%)
Internist	48 (15.6%)
Adult endocrinologist	17 (5.5%)
Pediatric endocrinologist	27 (8.8%)
Family medicine	82 (26.7%)
Others	57 (18.6%)
Current position	
Consultant	103 (33.6%)
Specialist/Registrar	58 (18.9%)
Resident	146 (47.6%)
Years in practice	
1–5 years	163 (53.1%)
6–10 years	68 (22.1%)
11–20 years	52 (16.9%)
>20 years	24 (7.8%)
Presence of endocrinologist	
Yes	259 (84.4%)
No	48 (15.6%)

**Table 2:** Assessment of the knowledge regarding insulin pump therapy (n = 307).

Statement	Correct Answer N (%)	Incorrect Answer N (%)
Function of insulin pump therapy	30 (9.8%)	277 (90.2%)
2. Type of insulin used in the pump	146 (47.6%)	161 (52.4%)
3. The main type(s) of insulin doses in the pump	176 (57.3%)	131 (42.7%)
4. Insulin pump therapy is recommended for which type of diabetic patient	43 (14.0%)	264 (86.0%)
5. Insulin pump therapy can be used for which age group	267 (87.0%)	40 (13.0%)
6. Mechanism of loading insulin pump by the patient	143 (46.6%)	164 (53.4%)
7. The frequency of changing infusion set of insulin pump, who is responsible for this action.	121 (39.4%)	186 (60.6%)
8. The best candidate for insulin pump therapy	165 (53.7%)	142 (46.3%)
9. Insulin pumps come in different types	201 (65.5%)	106 (34.5%)
10. The patient needs to do very well with the new technology to be on insulin pump	88 (28.7%)	219 (71.3%)
11. The pump needs to be implanted, and therefore minor surgery is needed	149 (48.5%)	158 (51.5%)
12. The pump can be disconnected even for short time (<1 h).	147 (47.9%)	160 (52.1%)
13. Severe hyperglycemia or possibly diabetes ketoacidosis can results from pump discontinuation even for several hours	164 (53.4%)	143 (46.6%)
14. The pump (especially if attached to continuous glucose monitoring) eliminates the need for self(finger-stick) glucose monitoring	88 (28.7%)	219 (71.3%)

**Table 3:** Assessment of the attitude toward insulin pump therapy (n = 307).

Statement	SD N (%)	D N (%)	N N (%)	A N (%)	SA N (%)
1. Physicians should know the basic information and understand primary principles of insulin pump therapy	01 (0.30%)	07 (2.3%)	46 (15.0%)	104 (33.9%)	149 (48.5%)
2. Each hospital should have a structured diabetes program (start from assessment & education, to determination & initiation, then to outcome evaluation) for patients who are on insulin pump	02 (0.70%)	04 (1.3%)	16 (5.2%)	80 (26.1%)	205 (66.8%)
3. Insulin pump therapy promotes the patient emotionally & psychologically to improve the management of their high blood sugar	0	03 (1.0%)	25 (8.1%)	120 (39.1%)	159 (51.8%)
4. The selection of eligible candidates for insulin pump therapy depends more on the patient motivation and readiness than desires by physicians or family	0	12 (3.9%)	49 (16.0%)	122 (39.7%)	124 (40.4%)
5. The major barrier to insulin pump therapy is the high cost more than the associated safety issues or adverse effects	02 (0.70%)	45 (14.7%)	76 (24.8%)	124 (40.4%)	60 (19.5%)
6. Educational programs for diabetic patients about the benefits & risk of insulin pump therapy are needed	0	0	04 (1.3%)	95 (30.9%)	208 (67.8%)

SD—Strongly Disagree; D—Disagree; N—Neutral; A—Agree; SA—Strongly Agree.

**Table 4:** Comparison of the mean knowledge and attitude scores according to the physicians’ sociodemographic characteristics (n = 307).

Factor	Knowledge		Attitude	
	Score (14) Mean ± SD	*p*-Value	Score (30) Mean ± SD	*p*-Value
Age group ^a^				
21–30 years	4.94 ± 2.82	*p* < 0.001 **	25.4 ± 2.63	*p* = 0.049 **
31–40 years	7.71 ± 3.49		25.9 ± 2.55	
>40 years	7.60 ± 3.23		26.4 ± 2.49	
Gender ^b^				
Male	6.43 ± 3.46	*p* = 0.212	25.7 ± 2.56	*p* = 0.425
Female	5.85 ± 3.21		25.9 ± 2.75	
Nationality ^b^				
Saudi	6.21 ± 3.41	*p* = 0.173	25.7 ± 2.62	*p* = 0.425
Non-Saudi	7.25 ± 3.23		26.2 ± 2.37	
Residence region ^b^				
Inside Central Region	6.29 ± 3.53	*p* = 0.984	25.5 ± 2.69	*p* = 0.121
Outside Central region	6.27 ± 3.24		26.0 ± 0.48	
Specialty ^a^				
Pediatrician	5.29 ± 2.75	*p* < 0.001 **	25.9 ± 2.49	*p* = 0.863
Internist	6.25 ± 3.03		25.9 ± 2.53	
Adult endocrinologist	9.76 ± 2.36		25.9 ± 2.91	
Pediatric endocrinologist	10.7 ± 1.56		26.1 ± 2.53	
Family medicine	5.84 ± 3.42		25.8 ± 2.71	
Others	5.11 ± 3.06		25.0 ± 2.57	
Current position ^a^				
Consultant	7.79 ± 3.71	*p* < 0.001 **	26.0 ± 2.63	*p* = 0.256
Specialist/Registrar	7.53 ± 2.87		26.0 ± 2.65	
Resident	4.71 ± 2.59		25.4 ± 2.55	
Years in practice ^a^				
1–5 years	5.09 ± 2.88	*p* < 0.001 **	25.5 ± 2.63	*p* = 0.114
6–10 years	7.46 ± 3.51		25.7 ± 2.79	
>10 years	7.76 ± 3.51		26.3 ± 2.32	
Presence of endocrinologist ^b^				
Yes	6.44 ± 3.39	*p* = 0.071	25.8 ± 2.62	*p* = 0.149
No	5.39 ± 3.31		25.4 ± 2.54	
Non-endocrinologist who had seen insulin pump at current workplace with/without the presence of endocrinologist (n = 144)				
Presence of endocrinologist	6.67 ± 2.89	*p* = 0.473	26.1 ± 2.55	*p* = 0.197
Absence of endocrinologist	7.14 ± 2.76		25.3 ± 2.69	
Non-endocrinologist who had seen patients with insulin pump (n = 263)				
Never	4.22 ± 2.81	*p* < 0.001 **	25.3 ± 2.58	*p* = 0.051
Once per year	6.68 ± 2.63		26.0 ± 2.55	
2–3 times per year	6.86 ± 2.48		25.7 ± 2.74	
>3 times per year	6.18 ± 2.82		27.6 ± 2.42	
Once per month	6.10 ± 2.36		25.9 ± 2.58	
2–3 times per month	7.61 ± 3.84		25.7 ± 2.34	
>3 times per month	7.29 ± 2.56		27.0 ± 3.16	

^a^*p*-value has been calculated using Kruskal–Wallis test. ^b^*p*-value has been calculated using Mann–Whitney U test. ** Significant at *p* < 0.05 level.

## Supplementary Materials

The following are available online at https://www.mdpi.com/1660-4601/17/24/9394/s1, Table S1: Post Hoc Analysis for the knowledge score (*n* = 307), Table S2: Post Hoc Analysis for the knowledge score (cont’d.) (*n* = 307), Table S3: Post Hoc Analysis for the knowledge score (cont’d.), Table S4: Post Hoc Analysis for the attitude score (*n* = 307).
