# Supplementary material for: Are Physicians in Saudi Arabia Ready for Patients with an Insulin Pump? An Examination of Physician Knowledge and Attitude
Source: Int J Environ Res Public Health. 2020 Dec 15;17(24):9394. doi: 10.3390/ijerph17249394 (PMC7765397; doi:10.3390/ijerph17249394)
Supplement: Supplementary file 1 [file ijerph-17-09394-s001.pdf]

Supplementary Tables

Table S1. Post Hoc Analysis for the knowledge score (n=307).

Age Group (I)	Age Group (J)	Mean Diff. (I-J)	Std. Error	P-value §
21–30 years	31–40 years	-2.768	0.391	<0.001 **
	>40 years	2.266	0.537	<0.001 **
31–40 years	21–30 years	2.768	0.391	<0.001 **
	>40 years	0.106	0.564	1.000
>40 years	21–30 years	2.662	0.537	<0.001 **
	31–40 years	-0.106	0.564	1.000
**Specialty (I)**	**Specialty (J)**			
Pediatrician	Internist	-0.961	0.544	1.000
	Adult endocrinologist	-4.475	0.792	<0.001 **
	Pediatric endocrinologist	-5.451	0.662	<0.001 **
	Family medicine	-0.552	0.470	1.000
	Others	0.184	0.517	1.000
Internist	Pediatrician	0.961	0.544	1.000
	Adult endocrinologist	-3.515	0.833	<0.001 **
	Pediatric endocrinologist	-4.491	0.710	<0.001 **
	Family medicine	0.409	0.537	1.000
	Others	1.145	0.579	0.731
Adult endocrinologist	Internist	3.515	0.833	<0.001 **
	Pediatrician	4.475	0.792	<0.001 **
	Pediatric endocrinologist	-0.976	0.914	1.000
	Family medicine	3.923	0.787	<0.001 **
	Others	4.659	0.816	<0.001 **
Pediatric endocrinologist	Pediatrician	5.451	0.662	<0.001 **
	Internist	4.491	0.710	<0.001 **
	Adult endocrinologist	0.976	0.914	1.000
	Family medicine	4.899	0.655	<0.001 **
	Others	5.635	0.689	<0.001 **
Family medicine	Pediatrician	0.552	0.470	1.000
	Internist	-0.409	0.537	1.000
	Adult endocrinologist	-3.923	0.787	<0.001 **
	Pediatric endocrinologist	-4.899	0.655	<0.001 **
	Others	0.736	0.509	1.000
Others	Pediatrician	-1.184	0.517	1.000
	Internist	-1.145	0.579	0.731
	Adult endocrinologist	-4.659	0.816	<0.001 **
	Pediatric endocrinologist	-5.635	0.689	<0.001 **

	Family medicine	-0.736	0.509	1.000
**Current position (I)**	**Current Position (J)**			
Consultant	Specialist/Registrar	0.262	0.503	1.000
	Resident	3.084	0.394	<0.001 **
Specialist/Registrar	Consultant	-0.262	0.503	1.000
	Resident	2.822	0.475	<0.001 **
Resident	Consultant	-3.084	0.394	<0.001 **
	Specialist/Registrar	-2.822	0.475	<0.001 **

§ P-value has been calculated using Bonferroni test. ** The mean difference is significant at the 0.05 level.

Table S2. Post Hoc Analysis for the knowledge score (cont'd.) (n=307).

Years in practice (I)	Years in practice (J)	Mean Diff. (I-J)	Std. Error	P-value §
1–5 years	6–10 years	-2.358	0.457	<0.001 **
	>10 years	-2.665	0.439	<0.001 **
6–10 years	1–5 years	2.358	0.457	<0.001 **
	>10 years	-0.307	0.529	1.000
>10 years	1–5 years	2.665	0.439	<0.001 **
	6–10 years	0.307	0.529	1.000
**Non-endocrinologist who had seen patients with insulin pump (I)**	**Non-endocrinologist who had seen patients with insulin pump (J)**			
Never	Once per year	-2.458	0.552	<0.001 **
	2–3 times per year	-2.639	0.671	0.002 **
	>3 times per year	-1.963	0.894	0.609
	Once per month	-1.882	0.518	0.007 **
	2–3 times per month	-3.394	0.572	<0.001 **
	>3 times per month	-3.067	1.103	0.123
Once per year	Never	2.458	0.552	<0.001 **
	2–3 times per year	-0.181	0.787	1.000
	>3 times per year	0.495	0.984	1.000
	Once per month	0.576	0.662	1.000
	2–3 times per month	-0.936	0.705	1.000
	>3 times per month	-0.609	1.177	1.000
2–3 times per year	Never	2.639	0.671	0.002 **
	Once per month	0.181	0.787	1.000
	>3 times per year	0.675	1.056	1.000
	Once per month	0.757	0.765	1.000
	2–3 times per year	-0.756	0.802	1.000
	>3 times per month	-0.429	1.238	1.000
>3 times per year	Never	1.963	0.894	0.609
	Once per year	-0.495	0.984	1.000
	>3 times per year	-0.675	1.056	1.000
	Once per month	0.082	0.966	1.000
	2–3 times per month	-1.431	0.996	1.000
	>3 times per month	-1.104	1.372	1.000
Once per month	Never	1.882	0.519	0.007 **
	Once per year	-0.576	0.662	1.000
	2–3 times per year	-0.757	0.765	1.000
	>3 times per year	-0.082	0.966	1.000
	2–3 times per month	-1.513	0.679	1.000
	>3 times per month	-1.186	1.162	1.000
2–3 times per month	Never	3.394	0.572	<0.001 **
	Once per year	0.936	0.705	1.000
	2–3 times per year	0.756	0.802	1.000
	>3 times per year	1.431	0.996	1.000
	Once per month	1.513	0.679	0.561
	>3 times per month	0.327	1.187	1.000
>3 times per month	Never	3.067	1.103	0.123
	Once per year	0.609	1.177	1.000

	2–3 times per year	0.429	1.238	1.000
	>3 times per year	1.104	1.372	1.000
	Once per month	1.186	1.162	1.000
	>3 times per month	-0.327	1.187	1.000

§ P-value has been calculated using Bonferroni test. ** The mean difference is significant at the 0.05 level.

Table S3. Post Hoc Analysis for the knowledge score (cont'd.).

Consultant (I)	Consultant (J)	Mean Diff. (I-J)	Std. Error	P-value §
Pediatrician	Internist	-1.018	1.184	1.000
	Adult endocrinologist	-4.642	1.051	<0.001 **
	Pediatric endocrinologist	-5.559	0.821	<0.001 **
	Family medicine	-3.509	0.945	0.005 **
	Others	-0.349	0.908	1.000
Internist	Pediatrician	1.018	1.184	1.000
	Adult endocrinologist	-3.625	1.372	0.144
	Pediatric endocrinologist	-4.541	1.205	0.004 **
	Family medicine	-2.491	1.292	0.852
	Others	0.669	1.266	1.000
Adult endocrinologist	Internist	4.643	1.051	<0.001 **
	Pediatrician	3.625	1.372	0.144
	Pediatric endocrinologist	-0.917	1.075	1.000
	Family medicine	1.133	1.172	1.000
	Others	4.294	1.142	0.004 **
Pediatric endocrinologist	Pediatrician	5.559	0.821	<0.001 **
	Internist	4.541	1.205	0.004 **
	Adult endocrinologist	0.917	1.075	1.000
	Family medicine	2.050	0.972	0.562
	Others	5.211	0.936	<0.001 **
Family medicine	Pediatrician	3.509	0.945	0.005 **
	Internist	2.492	1.292	0.852
	Adult endocrinologist	-1.133	1.172	1.000
	Pediatric endocrinologist	-2.050	0.972	0.562
	Others	3.161	1.046	0.048 **
Others	Pediatrician	0.349	0.908	1.000
	Internist	-0.669	1.266	1.000
	Adult endocrinologist	-4.294	1.142	0.004 **
	Pediatric endocrinologist	-5.211	0.936	<0.001 **
	Family medicine	-3.161	1.046	0.048 **

§ P-value has been calculated using Bonferroni test. ** The mean difference is significant at the 0.05 level.

Table 4. Post Hoc Analysis for the attitude score (n=307).

Age Group (I)	Age Group (J)	Mean Diff. (I-J)	Std. Error	P-value §
21–30 years	31–40 years	-0.599	0.324	0.196
	>40 years	-1.013	0.445	0.070
31–40 years	21–30 years	0.599	0.324	0.196
	>40 years	-0.414	0.467	1.000
>40 years	21–30 years	1.013	0.445	0.070
	31–40 years	0.414	0.467	1.000

§ P-value has been calculated using Bonferroni test.
